# Supplementary material for: High-resolution analysis of Merkel Cell Polyomavirus in Merkel Cell Carcinoma reveals distinct integration patterns and suggests NHEJ and MMBIR as underlying mechanisms
Source: PLoS Pathog. 2020 Aug 24;16(8):e1008562. doi: 10.1371/journal.ppat.1008562 (PMC7470373; doi:10.1371/journal.ppat.1008562)
Supplement: S1 Fig — Sequences at the virus-host junction (in grey) were derived from capture sequencing and aligned to reference sequences for the human genome (hg38) and MCPyV (JN707599). L = left side of the integrated viral genome, R = right side. Depicted are 40 bps upstream and downstream from the virus-host junction (indicated by a black line). In the case of insertions at the junction, sequences were extended for the length of the insertion. In the case of identical bases between virus and host sequences directly at the junction, both options are depicted including both homology scores (identical sequences to virus, identical sequences to host, respectively). Human sequences are depicted in blue and viral sequences in black letters. Detected microhomologies (see material and methods) are marked in red. (PDF) [file ppat.1008562.s001.pdf]

WaGa

R: hg38 (chr6:20,569,311)/ JN707599 (3516); **bold**=insertion from left integration site (chr6: 20,635,153-20,635,183), lowercase letters=insertion from unknown origin, scores: virus (30)/host (13)

chr6 (R): GCCTCACTATATTAATGGCCTTATTGGTGCTTTACAGATTTTGTGATTCATGGGCACTGTAAGTATAAATTTCATGCTTACAGCAATTGAATAAAATAGGCACATTGTTCTTTGTTA
MCPyV (R): GCAAAACCATTTCCTTGCCAAAAGTGTGAAAACAGATCTCGCCTCAAACCTCACAAGGCTCATGAGGCTCATCATTCTAATGCTAAGCTATTTTATGAATCTAAATCTCAGAAAACC
MCPyV/chr6: GCAAAACCATTTCCTTGCCAAAAGTGTGAAAACAGATCTC|a**TATTGTATTTCATAGGTATCCTTACCATTT**attcaa|TTACAGCAATTGAATAAAATAGGCACATTGTTCTTTGTTA
chr6 (L): GGTCTCAAATCCAATTCTCTGACCCTCTCAGTCCTGTTTTG**TATTGCATTTCATAGGTATCCTTACCATTT**GGCTTTTGTGAGTAGATCTGGACAATTGGAGGCACTGATGGAAGAC
chr6/MCPyV(L): GGTCTCAAATCCAATTCTCTGACCCTCTCAGTCCTGTTTTGTATTGCATT|TCACAGGTAATATCCTCATTTAGCATTGGCAGAGACACTCTTGCCACACTGTAAGCTGGCAAATTTT

L (identical bases to virus): hg38 (chr6: 20,635,162)/ JN707599 (1508); scores: host (25)/virus (46)

chr6: CCAATTCTCTGACCCTCTCAGTCCTGTTTTGTATTGCATTTCATAGGTATCCTTACCATTTGGCTTTTGTGAGTAGATCT
MCPyV: TTCTGTTTTAACAGATATTGCCTCCCACATCTGCAATGTGTCACAGGTAATATCCTCATTTAGCATTGGCAGAGACACTC
chr6/MCPyV: CCAATTCTCTGACCCTCTCAGTCCTGTTTTGTATTGCATT|TCACAGGTAATATCCTCATTTAGCATTGGCAGAGACACTC

L (identical bases to host): hg38 (chr6: 20,635,165)/ JN707599 (1505); scores: host (32)/ virus (39)

chr6: ATTCTCTGACCCTCTCAGTCCTGTTTTGTATTGCATTTCATAGGTATCCTTACCATTTGGCTTTTGTGAGTAGATCTGGA
MCPyV: TGTTTTAACAGATATTGCCTCCCACATCTGCAATGTGTCACAGGTAATATCCTCATTTAGCATTGGCAGAGACACTCTTG
chr6/MCPyV: ATTCTCTGACCCTCTCAGTCCTGTTTTGTATTGCATTTCA|CAGGTAATATCCTCATTTAGCATTGGCAGAGACACTCTTG

MKL-1

L (identical bases to virus): hg38 (chr5: 52,562,625)/ JN707599 (159); ~~crossed out~~=deleted between L and R junctions, scores: host (22)/virus (0)

chr5: GTGCAAGGAACCATATTGGATGTTGTAGAATAAGCCTAGA~~TCTA~~|TAGATAACTAATTTGTTTGATTTGGGGTAAGAAATT
MCPyV: TTTTTTGCTAATTAAGCCTCTTAAGCCTCAGAGGCCTCTCTCTTTTTTTTCCAGAGGCCTCGGAGGCTAGGAGCCCCAAG
chr5/MCPyV: GTGCAAGGAACCATATTGGATGTTGTAGAATAAGCCTAGA|TCTTTTTTTTCCAGAGGCCTCGGAGGCTAGGAGCCCCAAG

L (identical bases to host): hg38 (chr5: 52,562,628)/ JN707599 (162); ~~crossed out~~=deleted between L and R junctions, scores: host (30)/virus (0)

chr5: CAAGGAACCATATTGGATGTTGTAGAATAAGCCTAGATCTA|TAGATAACTAATTTGTTTGATTTGGGGTAAGAAATTAAA
MCPyV: TTTGCTAATTAAGCCTCTTAAGCCTCAGAGGCCTCTCTCTTTTTTTTCCAGAGGCCTCGGAGGCTAGGAGCCCCAAGCCT
chr5/MCPyV: CAAGGAACCATATTGGATGTTGTAGAATAAGCCTAGATCT|TTTTTTTCCAGAGGCCTCGGAGGCTAGGAGCCCCAAGCCT

R: hg38 (chr5: 52,562,630)/ JN707599 (1498); ~~crossed out~~=deleted between L and R junctions, underlined=duplicated from JN707599, **bold**=possible insertion from chr5: 52,562,618-52,562,621, scores: virus (8)/host (28)

chr5: AAATACCCAGGTGCAAGGAACCATATTGGATGTTGTAGAATA**AGCC**TAGA|~~TCTA~~TAGATAACTAATTTGTTTGATTTGGGGTAAGAAATTAAAT
MCPyV: AGTGTGGCAAGAGTGTCTCTGCCAATGCTAAATGAGGATATTACCTGTGACACATTGCAGATGTGGGAGGCAATATCTGTTAAAACAGAAGTAG
MCPyV/chr5: AGTGTGGCAAGAGTGTCTCTGCCAATGCTAAATGAGGATAAATGAGGATA**AGCC**|TAGATAACTAATTTGTTTGATTTGGGGTAAGAAATTAAAT

BroLi

L (identical bases to virus): hg38 (chr1: 10,790,267)/ JN707599 (543); ~~crossed out~~=deleted between L and R junctions, scores: host (26)/virus (26)

chr1: GGGGTGCTGGCAATGATGCTTCCAGCCTGCACCCTCCCAA~~CCACGCCACGTATCCCG~~|GGCACAGGGACCGGCCTTATCGG
MCPyV: GCTTCCAAAGCTGCTAAAGCTTCTCCTGTAAGAATAGCTTCCAAAGTTACTCCTGTGGTGGCACTTAGTTCAGTAGCAAT
chr1/MCPyV: GGGGTGCTGGCAATGATGCTTCCAGCCTGCACCCTCCCAA|CCAAAGTTACTCCTGTGGTGGCACTTAGTTCAGTAGCAAT

L (identical bases to host): hg38 (chr1: 10,790,270)/ JN707599 (540); ~~crossed out~~=deleted between L and R junctions, scores: host (34)/virus (14)

chr1: GTGCTGGCAATGATGCTTCCAGCCTGCACCCTCCCAACCA~~CGCCACGTATCCCG~~|GGCACAGGGACCGGCCTTATCGGTCT
MCPyV: TCCAAAGCTGCTAAAGCTTCTCCTGTAAGAATAGCTTCCAAAGTTACTCCTGTGGTGGCACTTAGTTCAGTAGCAATTTC
chr1/MCPyV: GTGCTGGCAATGATGCTTCCAGCCTGCACCCTCCCAACCA|AAGTTACTCCTGTGGTGGCACTTAGTTCAGTAGCAATTTC

R: hg38 (chr1: 10,790,285)/ JN707599 (4054); scores: virus (29)/host (12)

chr1: GCTTCCAGCCTGCACCCTCCCAA|~~CCACGCCACGTATCCCG~~GGCACAGGGACCGGCCTTATCGGTCTGCCGGGCTGCCCTT
MCPyV: AAGCTCTGCTAGTTCAGCAAGTTTTACAAGCACTCCACCAAAGCCAAAAAAGAACAGAGAAACTCCTGTTCCTACTGATT
MCPyV/chr1: AAGCTCTGCTAGTTCAGCAAGTTTTACAAGCACTCCACCA|GGCACAGGGACCGGCCTTATCGGTCTGCCGGGCTGCCCTT

LoKe

R: hg38 (chr2: 197,314,282)/ JN707599 (1802); lower case letters=insertion from unknown origin, scores: virus (32)/host (19)

chr2: TGACACTGCTTCTGAATTAATGCTAATTCCTGAGGATTGCAAACACCACTGGATCCACCAATCAAAATGGGGATCTATGGTAG
MCPyV: TCTATAGGATAATTTCCATCTTTATCTAATTTTGCTTTAGCTTGTGGATCTAGGCCCTGATTTTTAGGTGTCATTTTTCTTTCC
MCPyV/chr2: TCTATAGGATAATTTCCATCTTTATCTAATTTTGCTTTAGatc|CACCACTGGATCCACCAATCAAAATGGGGATCTATGGTAG

L (identical bases to virus): hg38 (chr2: 197,433,173)/ JN707599 (1811); scores: host (32)/virus (32)

chr2: CAACAAGCTATGTCAGTATTATGTGTATTTCTACTAAACATTTATCATCCCCTGACATATCCCATAGGGATGGGCACAGA
MCPyV: ACACCATACTTCTATAGGATAATTTCCATCTTTATCTAATTTTGCTTTAGCTTGTGGATCTAGGCCCTGATTTTTAGGTG
chr2/MCPyV: CAACAAGCTATGTCAGTATTATGTGTATTTCTACTAAACA|TTTGCTTTAGCTTGTGGATCTAGGCCCTGATTTTTAGGTG

L (identical bases to host): hg38 (chr2: 197,433,176)/ JN707599 (1808); scores: host (34)/ virus (19)

chr2: CAAGCTATGTCAGTATTATGTGTATTTCTACTAAACATTTATCATCCCCTGACATATCCCATAGGGATGGGCACAGACAG
MCPyV: CCATACTTCTATAGGATAATTTCCATCTTTATCTAATTTTGCTTTAGCTTGTGGATCTAGGCCCTGATTTTTAGGTGTCA
chr2/MCPyV: CAAGCTATGTCAGTATTATGTGTATTTCTACTAAACATTT|GCTTTAGCTTGTGGATCTAGGCCCTGATTTTTAGGTGTCA

MKL-2

R (identical bases to virus): hg38 (chr11: 62,505,277)/ JN707599 (640); scores: virus (30)/host (21)

chr11:	TGTCCCACGCCTCCAACCCAGTCTGCGGGCACATGGCTCACAGCAACCCCAGGGGATAAAAATCTGCCTAGCAACGGGTC
MCPyV:	TGACAATTGAGGGTATTTCTGGCATTGAGGCTTTAGCTCAACTTGGGTTCACAGCTGAACAGTTTTCAAATTTCTCATTA
MCPyV/chr11:	TGACAATTGAGGGTATTTCTGGCATTGAGGCTTTAGCTCA|CAGCAACCCCAGGGGATAAAAATCTGCCTAGCAACGGGTC

R (identical bases to host): hg38 (chr11: 62,505,272)/ JN707599 (635); scores: virus (16)/host (36)

chr11:	CTCTGTGTCCCACGCCTCCAACCCAGTCTGCGGGCACATGGCTCACAGCAACCCCAGGGGATAAAAATCTGCCTAGCAAC
MCPyV:	TTTAATGACAATTGAGGGTATTTCTGGCATTGAGGCTTTAGCTCAACTTGGGTTCACAGCTGAACAGTTTTCAAATTTCT
MCPyV/chr11:	TTTAATGACAATTGAGGGTATTTCTGGCATTGAGGCTTTA|GCTCACAGCAACCCCAGGGGATAAAAATCTGCCTAGCAAC

L: hg38 (chr11: 62,728,189)/ JN707599 (647); scores: host (30)/virus (13)

chr11:	AGATACCAAACTGTACGCATCTCTGACCCTTTCCTCTCCTTGCTCCTGCTGGTAAACCGAAGCCCAGGAGACTTCCAGGT
MCPyV:	TTGAGGGTATTTCTGGCATTGAGGCTTTAGCTCAACTTGGGTTCACAGCTGAACAGTTTTCAAATTTCTCATTAGTGGCT
chr11/MCPyV:	AGATACCAAACTGTACGCATCTCTGACCCTTTCCTCTCCT|GTTCACAGCTGAACAGTTTTCAAATTTCTCATTAGTGGCT

PeTa

L (identical bases to virus): hg38 (chr11: 25,063,416)/ JN707599 (706); 475-706 from JN707599 is inverted and fused to 4115 of JN707599 at the integration site, ~~crossed out~~=deleted between L and R junctions, scores: host (11)/virus (26)

chr11:	ATATTTAAAGCCATTTATCCATCTTAATACAATATTTTCC~~AAAATATCTGTGTGAATA~~|[R]TATCTTCACACCAGCATTGTAA
MCPyV (RC):	GCCTATACCACTAACAGTTTGGAGAATGAAGCCATAAGTTAAACCTTGGTTAACCAAAGAAGCCACTAATGAGAAATTTG
chr11/MCPyV:	ATATTTAAAGCCATTTATCCATCTTAATACAATATTTTCC|AAACCTTGGTTAACCAAAGAAGCCACTAATGAGAAATTTG

L (identical bases to host): hg38 (chr11: 25,063,419)/ JN707599 (703); 475-703 from JN707599 is inverted and fused to 4115 of JN707599 at the integration site, ~~crossed out~~=deleted between L and R junctions, scores: host (20)/virus (17)

chr11:	TTTAAAGCCATTTATCCATCTTAATACAATATTTTCCAAA~~ATATCTGTGTGAATA~~|[R]TATCTTCACACCAGCATTGTAAAAG
MCPyV (RC):	TATACCACTAACAGTTTGGAGAATGAAGCCATAAGTTAAACCTTGGTTAACCAAAGAAGCCACTAATGAGAAATTTGAAA
chr11/MCPyV:	TTTAAAGCCATTTATCCATCTTAATACAATATTTTCCAAA|CCTTGGTTAACCAAAGAAGCCACTAATGAGAAATTTGAAA

R: hg38 (chr11: 25,063,435)/ JN707599 (592); scores: virus (17)/host (23)

chr11:	CCATCTTAATACAATATTTTCC|[L]~~AAAATATCTGTGTGAATA~~TATCTTCACACCAGCATTGTAAAAGACAGAAAGGGAAAGA
MCPyV:	TTACAGGAGAAGCTTTAGCAGCTTTGGAAGCAGAGATCTCCAGTTTAATGACAATTGAGGGTATTTCTGGCATTGAGGCT
MCPyV/chr11:	TTACAGGAGAAGCTTTAGCAGCTTTGGAAGCAGAGATCTC|TATCTTCACACCAGCATTGTAAAAGACAGAAAGGGAAAGA

WoWe-2

R (identical bases to virus): hg38 (chr13: 71,480,943)/ JN707599 (1939); **bold**=TT insertion in host, scores: virus (20)/host (16)

chr13:	ATATGAAACAAGCTGAAGAAAGATTTCTCCGTGGCAGGTTGCCCCTCCCCACTCTTTTGTTTAACAGTAAAACATAGCTG
MCPyV:	GGCCCACTCCATTCTCATCTAAAAGGACAGTAGTTAGAGTATTACTAAATTGAAGAACTGTAGGAGTCTGAGAGCCTGTC
MCPyV/chr13:	GGCCCACTCCATTCTCATCTAAAAGGACAGTAGTTAGAGT|GCCCCTCCCCACTCTTT**TT**TGTTTAACAGTAAAACATAGCTG

R (identical bases to host): hg38 (chr13: 71,480,942)/ JN707599 (1940); **bold**=TT insertion in host, scores: virus (11)/host (9)

chr13:	GATATGAAACAAGCTGAAGAAAGATTTCTCCGTGGCAGGTTGCCCCTCCCCACTCTTTTGTTTAACAGTAAAACATAGCT
MCPyV:	GGGCCCACTCCATTCTCATCTAAAAGGACAGTAGTTAGAGTATTACTAAATTGAAGAACTGTAGGAGTCTGAGAGCCTGT
MCPyV/chr13:	GGGCCCACTCCATTCTCATCTAAAAGGACAGTAGTTAGAG|TGCCCCTCCCCACTCTTT**TT**TGTTTAACAGTAAAACATAGCT

L (identical bases to virus): hg38 (chr13: 71,546,428)/ JN707599 (1818); scores: host (18)/virus (24)

chr13:	AATAATGTATTTAATAAATGGCTCCTCCTGAAATATACCAATAAAATAATACTTAGTTATTACTATTTTGATATGAAATT
MCPyV:	GATCAGGACACCATACTTCTATAGGATAATTTCCATCTTTATCTAATTTTGCTTTAGCTTGTGGATCTAGGCCCTGATTT
chr13/MCPyV:	AATAATGTATTTAATAAATGGCTCCTCCTGAAATATACCA|ATCTAATTTTGCTTTAGCTTGTGGATCTAGGCCCTGATTT

L (identical bases to host): hg38 (chr13: 71,546,430)/ JN707599 (1816); scores: host (23)/virus (26)

chr13:	TAATGTATTTAATAAATGGCTCCTCCTGAAATATACCAATAAAATAATACTTAGTTATTACTATTTTGATATGAAATTAT
MCPyV:	TCAGGACACCATACTTCTATAGGATAATTTCCATCTTTATCTAATTTTGCTTTAGCTTGTGGATCTAGGCCCTGATTTTT
chr13/MCPyV:	TAATGTATTTAATAAATGGCTCCTCCTGAAATATACCAAT|CTAATTTTGCTTTAGCTTGTGGATCTAGGCCCTGATTTTT

UKE-MCC-1a

R: hg38 (chr9: 136,133,905)/ JN707599 (1654); lower case letters=insertion from unknown origin, scores: virus (32)/host (-9)

chr9:	CCCGGTTCCACCAGGATGTTCCACAGTCTCCTTCGGGGCTGGCAGCGAGGCCGAGGGGAGGAAGTGATTTGTCTCCGCGC
MCPyV:	AAACTAGGCCTTGCAAATCCAGAGGTTCTCCCCCAATGGCAAACATATGGTAATTTACCCCTGACACAGGAATACCAGCA
MCPyV/chr9:	AAACTAGGCCTTGCAAATCCAGAGGTTCTCCCCCAATGGCct|CAGCGAGGCCGAGGGGAGGAAGTGATTTGTCTCCGCGC

L (identical bases to virus): hg38 (chr9: 136,432,018)/ JN707599 (1663, MCPyV A) with insertion from a duplication in JN707599 (1372-1398, MCPyV B); underlined: duplication in JN707599, **bold**=insertion from duplication in JN707599, scores: host (17)/virus (23)

chr9:	ACGTCCGCTGCGGCACAGTGGGCCATGTGTGGGCACAGGCAGAGGGACGGCAGGTCCTTCCCCTTCCCCAGAACATGGCCTGGGAGTGGCTCTCAGGG
MCPyV A:	GTTTTTGGATACTCAGTCTGGTAATCTAAAACTAGGCCTTGCAAATCCAGAGGTTCTCCCCCAATGGCAAACATATGGTAATTTACCCCTGACACAGG
chr9/MCPyV:	ACGTCCGCTGCGGCACAGTGGGCCATGTGTGGGCACAGGC|**AGTATAAGTATAAGTATA**|CCCCAATGGCAAACATATGGTAATTTACCCCTGACACAGG
MCPyV B:	GTTTTGGCTGCAGGTCATAAGTATAAGTATACCAGTTTGA**AGTATAAGTATAAGTATA**CCAGTTTGAAGTAGTAGGAAGATCAGGGGAATTAACTCCC

L (identical bases to host): hg38 (chr9: 136,432,020)/ JN707599 (1663, MCPyV A) with insertion from a duplication in JN707599 (1372-1398, MCPyV B); underlined: duplication in JN707599, **bold**=insertion from duplication in JN707599, scores: host (16)/virus (24)

chr9: GTCCGCTGCGGCACAGTGGGCCATGTGTGGGCACAGGCAGAGGGACGGCAGGTCCTTCCCCTTCCCCAGAACATGGCCTGGGAGTGGCTCTCAGGG
MCPyV A: TTTTGGATACTCAGTCTGGTAATCTAAAACTAGGCCTTGCAAATCCAGAGGTTCTCCCCCAATGGCAAACATATGGTAATTTACCCCTGACACAGG
chr9/MCPyV: GTCCGCTGCGGCACAGTGGGCCATGTGTGGGCACAGGCAG|**TATAAGTATAAGTATA**|CCCCAATGGCAAACATATGGTAATTTACCCCTGACACAGG
MCPyV B: TTTGGCTGCAGGTCATAAGTATAAGTATACCAGTTTGAAG**TATAAGTATAAGTATA**CCAGTTTGAAGTAGTAGGAAGATCAGGGGAATTAACTCCC

UM-MCC-29

L (identical bases to virus): hg38 (chr5: 51,618,447)/ JN707599 (738); ~~crossed out~~=deleted between L and R junctions, scores: host (10)/virus (31)

chr5: GCTCAGACTGGGGAGGGCAGTGGAGGCAGGTGTCATGTTT~~CACAT~~|R AGGACTGAAATCACATAGTGAGTAAGGAGACACAC
MCPyV: CGTGACAACCTCACCCCCACAGTTATTAGAGAGCCTATACCACTAACAGTTTGGAGAATGAAGCCATAAGTTAAACCTTG
chr5/MCPyV: GCTCAGACTGGGGAGGGCAGTGGAGGCAGGTGTCATGTTT|CACTAACAGTTTGGAGAATGAAGCCATAAGTTAAACCTTG

L (identical bases to host): hg38 (chr5: 51,618,450)/ JN707599 (735); ~~crossed out~~=deleted between L and R junctions, scores: host (26)/virus (28)

chr5: CAGACTGGGGAGGGCAGTGGAGGCAGGTGTCATGTTTCAC~~AT~~|R AGGACTGAAATCACATAGTGAGTAAGGAGACACACAAA
MCPyV: GACAACCTCACCCCCACAGTTATTAGAGAGCCTATACCACTAACAGTTTGGAGAATGAAGCCATAAGTTAAACCTTGGTT
chr5/MCPyV: CAGACTGGGGAGGGCAGTGGAGGCAGGTGTCATGTTTCAC|TAACAGTTTGGAGAATGAAGCCATAAGTTAAACCTTGGTT

R (identical bases to virus): hg38 (chr5: 51,618,453)/ JN707599 (1519); scores: virus (26)/host (30)

chr5: GACTGGGGAGGGCAGTGGAGGCAGGTGTCATGTTT|L ~~CACAT~~AGGACTGAAATCACATAGTGAGTAAGGAGACACACAAATC
MCPyV: TTCCAACTACTTCTGTTTTAACAGATATTGCCTCCCACATCTGCAATGTGTCACAGGTAATATCCTCATTTAGCATTGGC
MCPyV/chr5: TTCCAACTACTTCTGTTTTAACAGATATTGCCTCCCACAT|AGGACTGAAATCACATAGTGAGTAAGGAGACACACAAATC

R (identical bases to host): hg38 (chr5: 51,618,448)/ JN707599 (1524); scores: virus (8)/host (36)

chr5: GCTCAGACTGGGGAGGGCAGTGGAGGCAGGTGTCATGTTT|L CACATAGGACTGAAATCACATAGTGAGTAAGGAGACACAC
MCPyV: ACTTATTCCAACTACTTCTGTTTTAACAGATATTGCCTCCCACATCTGCAATGTGTCACAGGTAATATCCTCATTTAGCA
MCPyV/chr5: ACTTATTCCAACTACTTCTGTTTTAACAGATATTGCCTCC|CACATAGGACTGAAATCACATAGTGAGTAAGGAGACACAC

UKE-MCC-4a

R I: hg38 (chr20: 31,665,275)/ JN707599 (4225); lower case letters=insertion from unknown origin, scores: virus (22)/host (24)

chr20: CTGAACTGCAAGGGCCACCCTAGCTTTGGCATGGGGTGCTTCCTGTAGCTATAGCCCCTCCCTCCTGGGTGGGGCTGGCCTC
MCPyV: TGTAAACTGAGATGACGAGGCCTCCTCGGCAGAGGAAGACGGGGGCTGCCGGGGCGAGCTTCTTGAGGAGGGGGGCTCCTCAG
MCPyV/chr20: TGTAAACTGAGATGACGAGGCCTCCTCGGCAGAGGAAGACcag|TGTAGCTATAGCCCCTCCCTCCTGGGTGGGGCTGGCCTC

L II (identical bases to virus): hg38 (chr20: 31,715,051)/ JN707599 (2261); #:60 reads; *:14 reads, lower case letter= mutation, scores: host (25)/virus (34)

chr20: CGCCTGTAATTCCAGCACTTTAGGAGGCTGAGGCGGGCGGATCACGAGGTCAGGAGATCGAGACCATCCTGACTAACATG
MCPyV-RC: AACAGTTTTCTCCTGCCCAAATTTATCTAAAAATCTGACAATATCAGGATCACCAGGTAATTGTTCTGACCCCTCATATA
#chr20/MCPyV-RC: CGCCTGTAATTCCAGCACTTTAGGAGGCTGAGGCGGGCGG|ATATCAGGATCACCAGGTAATTGTTCTGACCCCTCATATA
*chr20/MCPyV-RC: CGCCTGTAATTCCAGCACTTTAGGAGGCTGAGGCGGGCGG|gTATCAGGATCACCAGGTAATTGTTCTGACCCCTCATATA

L II (identical bases to host): hg38 (chr20: 31,715,053)/ JN707599 (2259); scores: host (23)/virus (30)

chr20: CCTGTAATTCCAGCACTTTAGGAGGCTGAGGCGGGCGGATCACGAGGTCAGGAGATCGAGACCATCCTGACTAACATGGT
MCPyV-RC: CAGTTTTCTCCTGCCCAAATTTATCTAAAAATCTGACAATATCAGGATCACCAGGTAATTGTTCTGACCCCTCATATATT
#chr20/MCPyV-RC: CCTGTAATTCCAGCACTTTAGGAGGCTGAGGCGGGCGGAT|ATCAGGATCACCAGGTAATTGTTCTGACCCCTCATATATT

R II: hg38 (chr20: 31,749,139)/ JN707599 (3755); lower case letters=insertion from unknown origin, scores: virus (31)/host (15)

chr20: CACCACCACGCCTGGCTAATTTTTTTGTATTTTTAGTAGAGACGGGGTTTCACTGTGTTAGCCAGGATGGTCTCAATCTCTTGAC
MCPyV-RC: GCTCTACCTTCTGCACTATAAGCTTTTTAATTTGTAAAGGAGTGAATAAGATGCCTGAAATGTATAATAATTTATGCAAGCCCCC
MCPyV-RC/chr20: GCTCTACCTTCTGCACTATAAGCTTTTTAATTTGTAAAGGgcgga|GGTTTCACTGTGTTAGCCAGGATGGTCTCAATCTCTTGAC

L I: hg38 (chr20: 31,785,438)/ JN707599 (397); lower case letters=insertion from unknown origin, scores: host (33)/virus (16)

chr20: GTTTAGTTTTACATACAAGATAATGGAGCCCCAGATAAGAGAAATTATTTCTTCAGGGTTTCTATAACACTCTTTTGGCACCTTGCTTTTGGT
MCPyV: GTTTGGGAGGGAGACGGAAGACTCTTAACTTTTTTTCAACAAGGGAGGCCCGGAGGCTTTTTTTTCTCTTACAAAGGGAGGAGGACATTAAAA
chr20/MCPyV: GTTTAGTTTTACATACAAGATAATGGAGCCCCAGATAAGA|taaaagccagataAGGCTTTTTTTTCTCTTACAAAGGGAGGAGGACATTAAAA

UM-MCC-52 Chr4

R: hg38 (chr4: 189,948,666)/ JN707599 (3916); lower case letters=insertion from unknown origin, scores: virus (26)/host (0)

chr4: TGATGCTAGAATGAGGGTGCCCTGGTACTATCTTATCAGCCATGACACTGGTGCATTGGGCCATTTTTTTTGTTTTGTTTTT
MCPyV: TTTAAAATCAACTTTAAATTTCTCAATCTTATCATATAACTCTATAGCTTTATCAGAAGTAGTATAAATGGCAAAACAACT
MCPyV/chr4: TTTAAAATCAACTTTAAATTTCTCAATCTTATCATATAACa|ATGACACTGGTGCATTGGGCCATTTTTTTTGTTTTGTTTTT

L: hg38 (chr4: 189,965,715)/ JN707599 (1136); lower case letters=insertion from unknown origin, scores: host (21)/virus (39)

chr4: GCCCTAACAGAGCTCCTGAAGGAAGCACTAAACATGGAAAGGAACAACCTGTACAAGCCACTGCAAAAACATGCCAAATT
MCPyV: TTCTTCCTCTGGTACTAGGCCTTAGTGGATATATTTCTCCTGAATTACAAGTAATTGAAGATGGCACCAAAAAGAAAAGC
chr4/MCPyV: GCCCTAACAGAGCTCCTGAAGGAAGCACTAAACATGGAAA|TGAcATTACAAGTAATTGAAGATGGCACCAAAAAGAAAAG

UM-MCC-52 Chr5

R (identical bases to virus): hg38 (chr5: 150,238,240)/ JN707599 (2470); scores: host (14)/virus (20)

chr5: AGCACTTTGGGAGGCCGAGGCAGGAGGATCACCTGGGGTCAGCAGTTGGAGACCAGCCTGGCCAACATGGTGAAACCCCA
MCPyV-RC: ATTATTTCATGCATTCCTATTCAGTTAAGTAGGCCCCAGAAAAACAAACACAGGAAATATGAAGCAGATGCCTTTATTGA
chr5/MCPyV-RC: AGCACTTTGGGAGGCCGAGGCAGGAGGATCACCTGGGGTC|AAAACAAACACAGGAAATATGAAGCAGATGCCTTTATTGA

R (identical bases to host): hg38 (chr5: 150,238,241)/ JN707599 (2471); scores: host (12)/virus (21)

chr5: GCACTTTGGGAGGCCGAGGCAGGAGGATCACCTGGGGTCAGCAGTTGGAGACCAGCCTGGCCAACATGGTGAAACCCCAT
MCPyV-RC: TTATTTCATGCATTCCTATTCAGTTAAGTAGGCCCCAGAAAAACAAACACAGGAAATATGAAGCAGATGCCTTTATTGAG
chr5/MCPyV-RC: GCACTTTGGGAGGCCGAGGCAGGAGGATCACCTGGGGTCA|AAACAAACACAGGAAATATGAAGCAGATGCCTTTATTGAG

L: hg38 (chr5: 150,404,900)/ JN707599 (1855); lower case letters=insertion from unknown origin, scores: host (9)/virus (11)

chr5: CAAATAGGGCCCCAACACCCGGGTCCAGCTGCAGCCTGGTTCCCTCTTAAAGACCCCTGGGGCTGAACCCCACATACTCTG
MCPyV: AATAGACCCATAGTATCTACTGTTTTCATTTTTAGAAGGATCAGGACACCATACTTCTATAGGATAATTTCCATCTTTATC
chr5/MCPyV: CAAATAGGGCCCCAACACCCGGGTCCAGCTGCAGCCTGGT|cCAGGACACCATACTTCTATAGGATAATTTCCATCTTTATC

MCC-47T and MCC-47M (primary tumor and metastasis)

R (identical bases to virus): hg38 (chr3: 64,619,639)/ JN707599 (5193); scores: virus (23)/host (8)

chr3: ATTAACAGGATAATAGATAATGGGATAATATAAGAACAATCCTATT|AGTAAAAGAATATTAATAGGATAATATATAACTG
MCPyV: GATGGAATTGAACACCCTTTGGAGCAAATTCCAGCAAAATATCCACAAGCTCAGAAGTGACTTCTCTATGTTTGATGAGG
MCPyV/chr3: GATGGAATTGAACACCCTTTGGAGCAAATTCCAGCAAAAT|CCTATTAGTAAAAGAATATTAATAGGATAATATATAACTG

R (identical bases to host): hg38 (chr3: 64,619,636)/ JN707599 (5196); scores: virus (16)/host (24)

chr3: TATATTAACAGGATAATAGATAATGGGATAATATAAGAACAATCCTATT|AGTAAAAGAATATTAATAGGATAATATATAA
MCPyV: AATGATGGAATTGAACACCCTTTGGAGCAAATTCCAGCAAAATATCCACAAGCTCAGAAGTGACTTCTCTATGTTTGATG
MCPyV/chr3: AATGATGGAATTGAACACCCTTTGGAGCAAATTCCAGCAA|AATCCTATTAGTAAAAGAATATTAATAGGATAATATATAA

L: hg38 (chr3: 64,619,644)/ JN707599 (5290); **bold**=duplicated from R junction, underlined=duplicated in host, scores: host (21)/virus (28)

chr3: AGGATAATAGATAATGGGATAATATAAGAACAAT|CCTATTAGTAAAAGAATATTAATAGGATAATATATAACTGTTAGAA
MCPyV: GCTCCTAATTGTTATGGCAACATCCCTCTGATGAAAGCTGCTTTCAAAAGAAGCTGCTTAAAGCATCACCCTGATAAAGG
chr3/MCPyV: ATGGGATAATATAAGAATATAATATAAGAACAAT**CCTATT**|CTTTCAAAAGAAGCTGCTTAAAGCATCACCCTGATAAAGG
