# Supplementary figures and images for: High-resolution analysis of Merkel Cell Polyomavirus in Merkel Cell Carcinoma reveals distinct integration patterns and suggests NHEJ and MMBIR as underlying mechanisms
Source: PLoS Pathog. 2020 Aug 24;16(8):e1008562. doi: 10.1371/journal.ppat.1008562 (PMC7470373; doi:10.1371/journal.ppat.1008562)

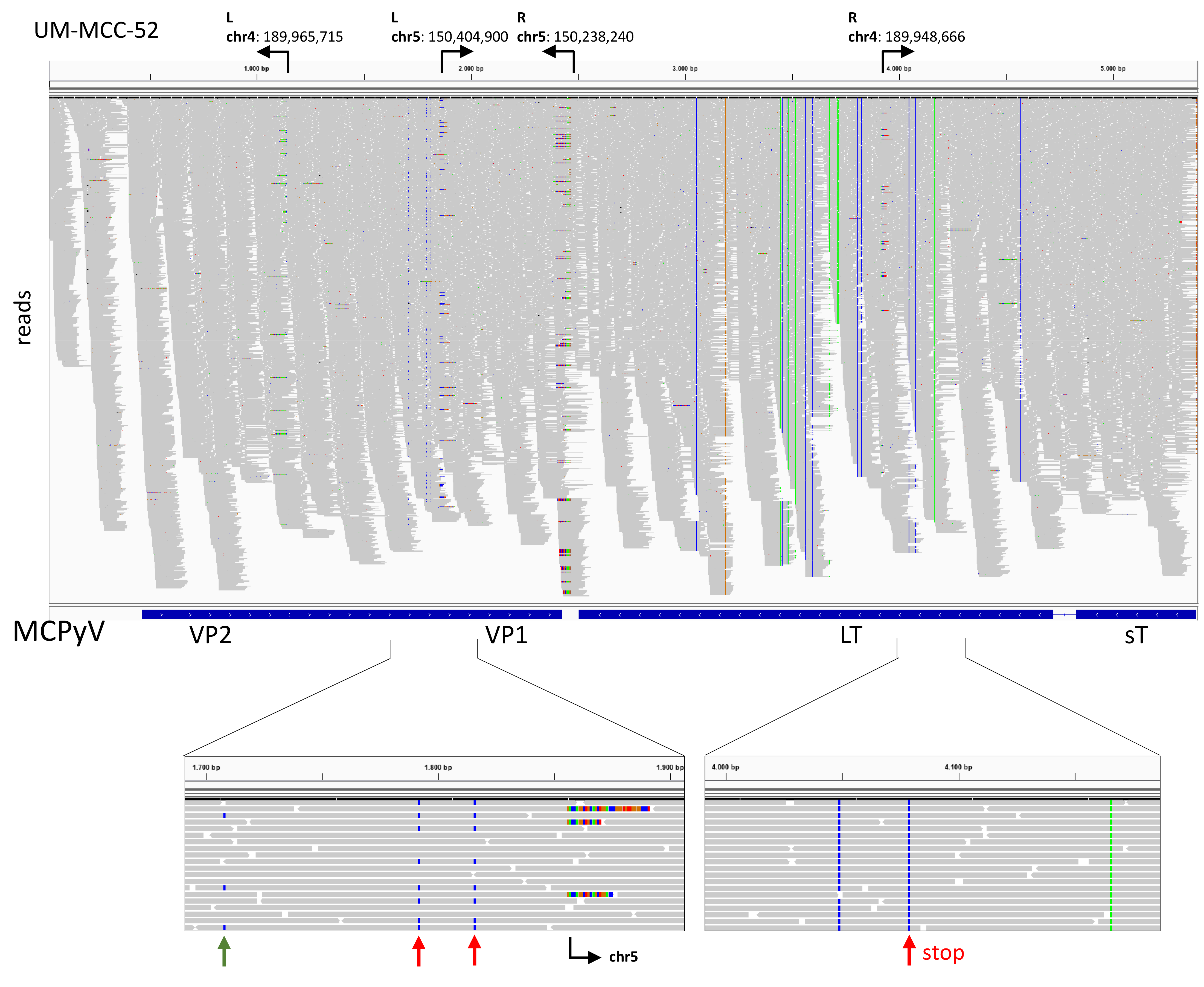

Supplement: S2 Fig — Grey color represents perfect matching of read and reference sequence. Blue, red, green and orange show mutations in the read sequence to the bases C, T, A and G respectively. Breakpoints into the host genome are indicated at the top reflected by longer stretches of mismatching bases. Lower panels show magnification of alignment. Mutations at bp 1,792 and 1,816 (G to C, left panel, red arrows) are not present in reads leading into Chr5. Reads that contain these mutations also contain a G to C transition at bp 1,708 (green arrow). Mutations in LT including the inactivating mutation (stop) are present in all captured sequences (right panel). (TIF) [file ppat.1008562.s002.tif]

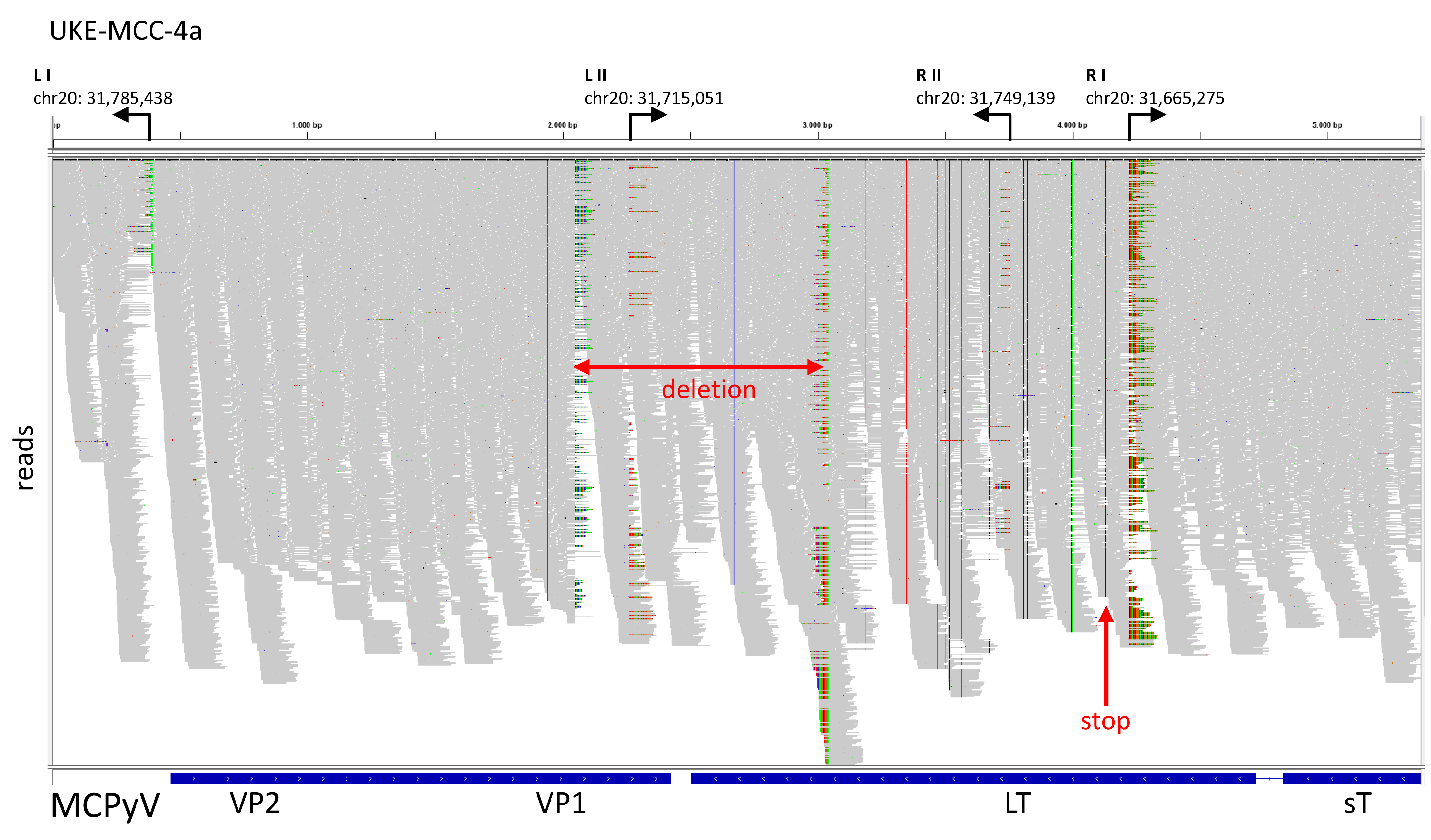

Supplement: S3 Fig — Color code is identical to S2 Fig. Breakpoints into the host genome are indicated at the top and can be recognized by longer stretches of mismatching bases. Bp 2,053 to 3,047 are deleted in approximately one third of the reads covering the region. This region also contains a breakpoint into the host genome indicating an integration of two versions of MCPyV (one with and one without a deletion). Mutations in LT including the inactivating mutation (stop) are present in all captured sequences. (TIF) [file ppat.1008562.s003.tif]

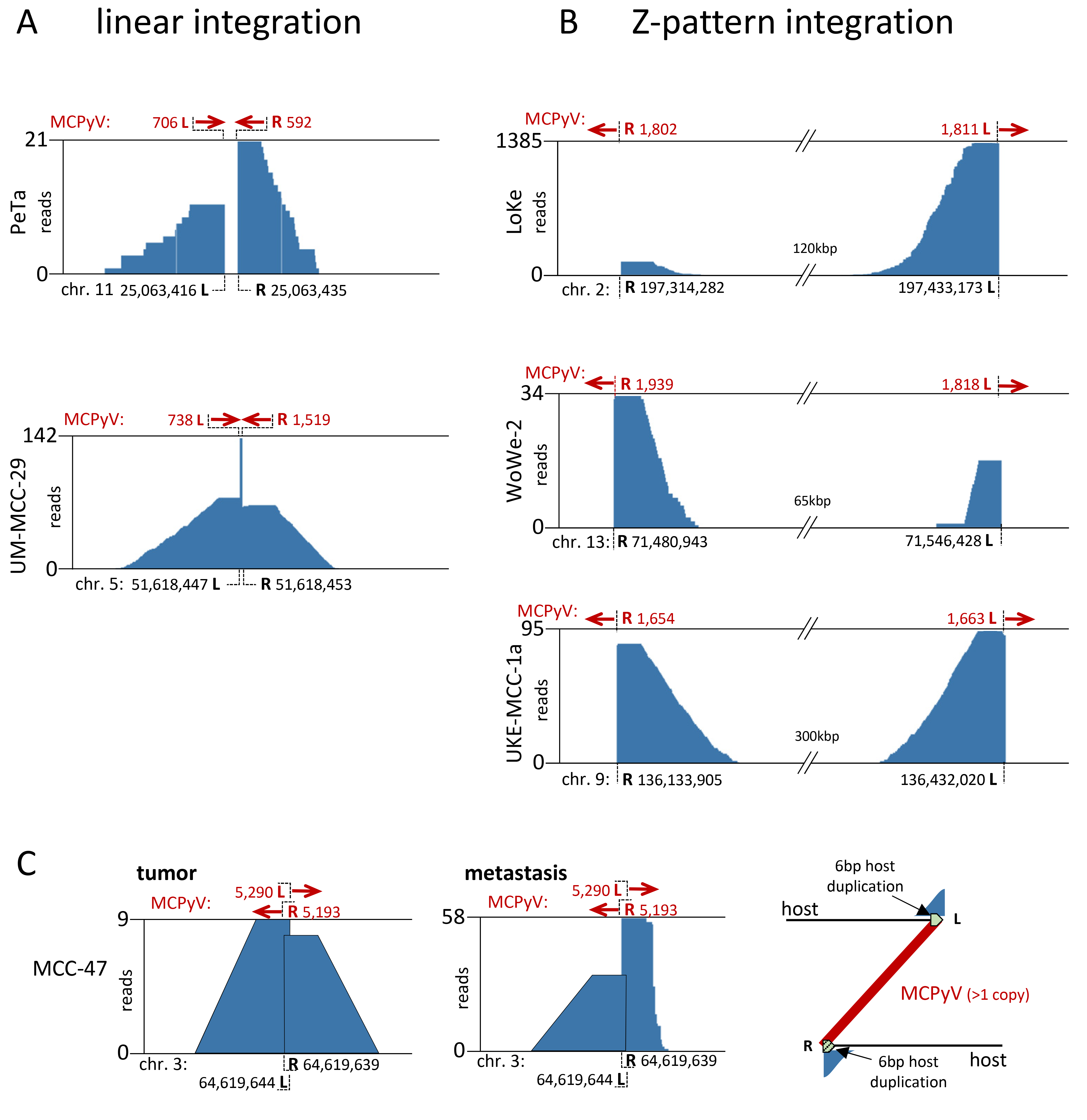

Supplement: S4 Fig — MCPyV-host fusion reads from capture sequencing were mapped to the human genome. (A): PeTa and UM-MCC-29 show a coverage profile characteristic for a linear integration pattern. (B): LoKe, WoWe-2 and UKE-MCC-1a show a coverage profile characteristic for a Z-pattern integration. (C): The sample MCC-47 (tumor and metastasis) shows a coverage profile with short distance (4bp) of breakpoints on the host genome but outward-facing orientation of viral sequences. The result is a Z-pattern integration with duplication of 6bp of host DNA as depicted in the right panel. Reads for both junctions of the tumor and the left junction of the metastasis are mapped by BLAST only. (TIF) [file ppat.1008562.s004.tif]

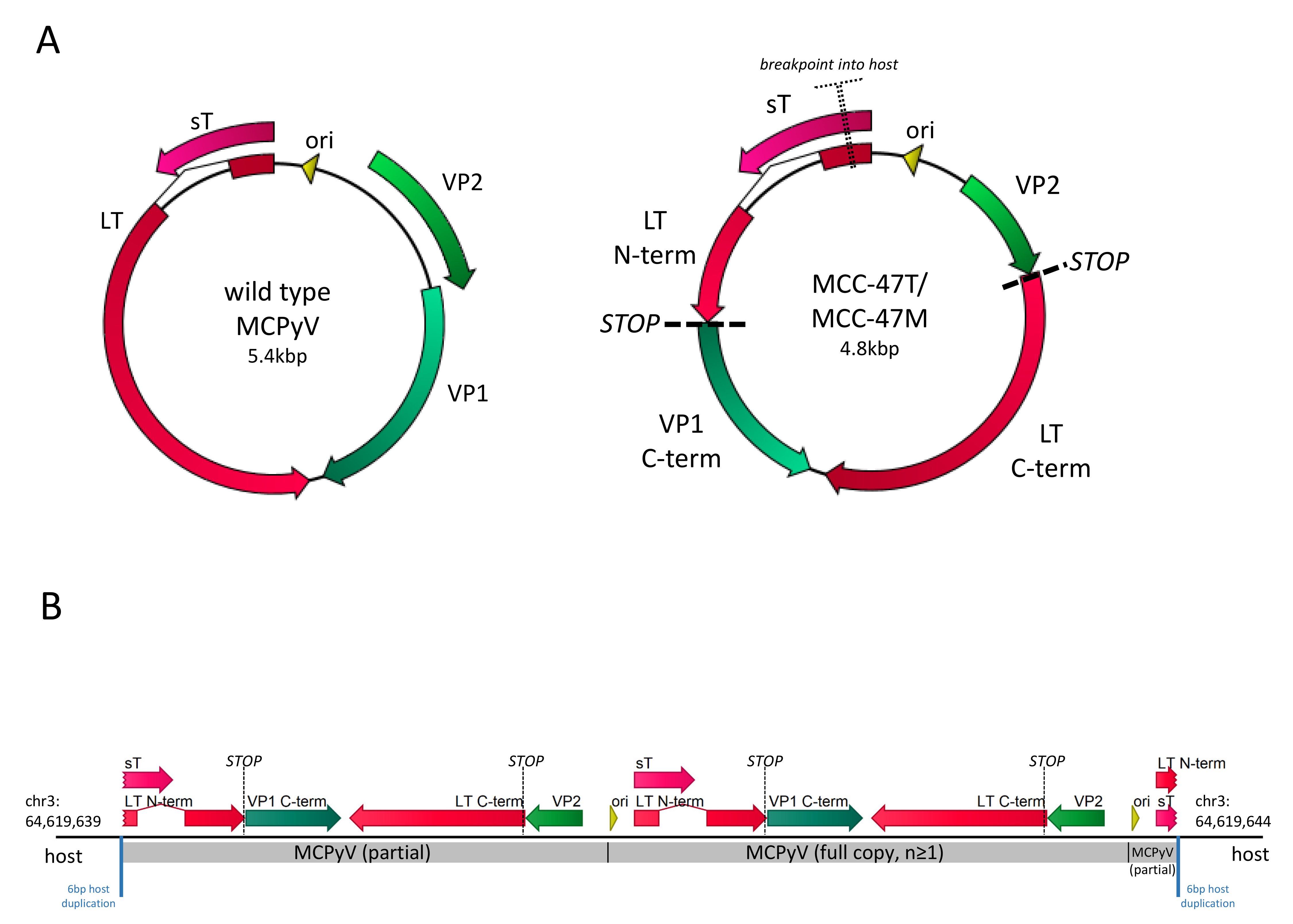

Supplement: S5 Fig — (A): Rearranged MCPyV genome derived from capture sequencing of sample MCC-47 (primary tumor and metastasis) compared to MCPyV wild type (JN707599). For better comparison, both genomes are depicted as episomes. Breakpoints into the host genome are indicated (bp 5,193 and 5,290). Bp 1547–4119 are inverted with 1,547 fused to 4,166 and 4,119 to 991 causing a frameshift in LT that leads to a stop at position 4,166. The C-terminal part of LT fused to VP2 is also out of frame, which causes a stop at the beginning of the LT C-terminus. (B): Integration locus of MCC-47 derived from capture sequencing (chr3: 64,619,639–44). The rearranged MCPyV genome is integrated as a concatemer with at least one complete viral genome being flanked by partial genomes that connect into the host genome. 6bp of host sequence are duplicated at the integration site. (TIF) [file ppat.1008562.s005.tif]

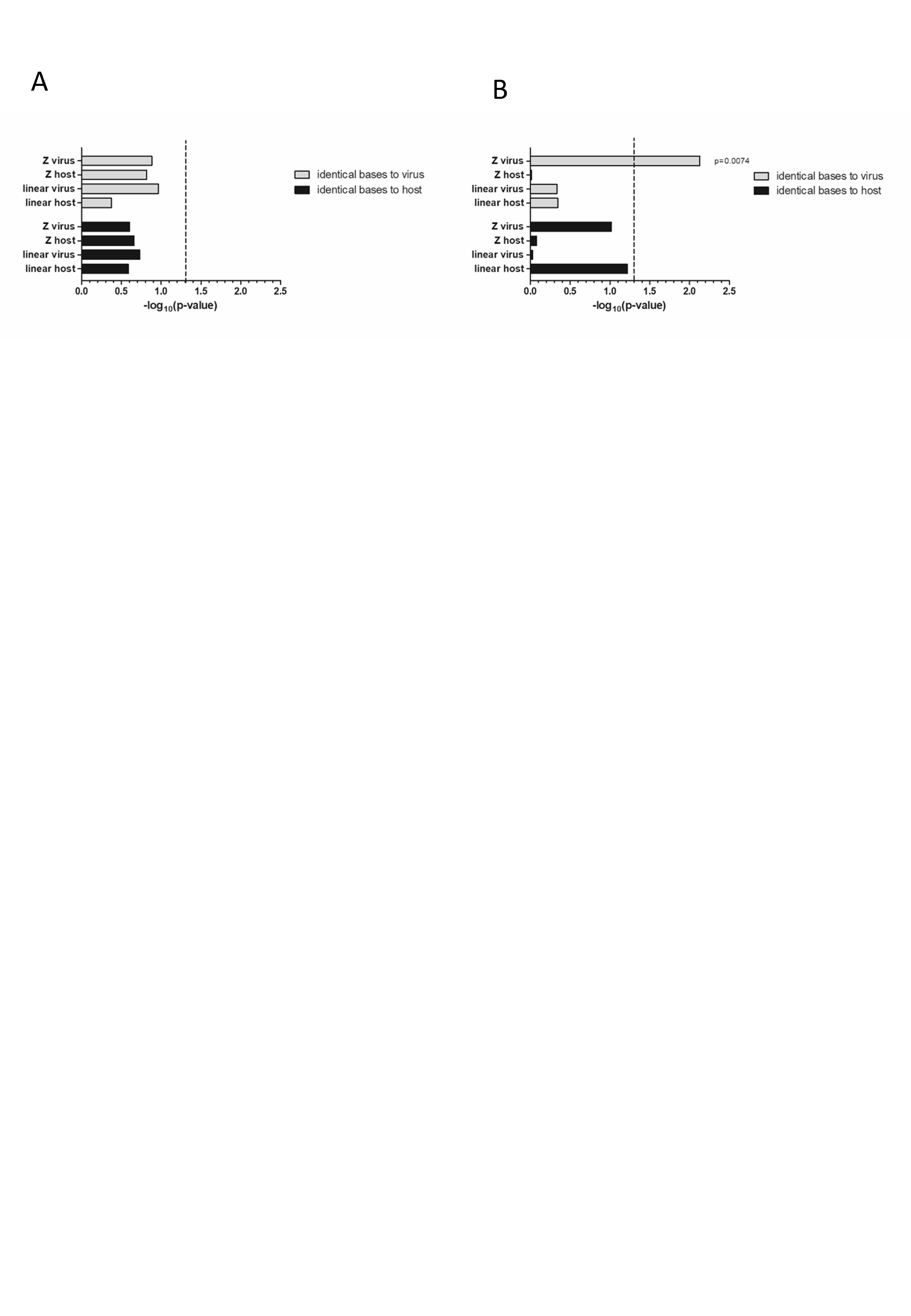

Supplement: S6 Fig — (A): Statistical analysis of global alignments between virus and host sequences at integration sites. 40bp of viral and host sequences from the virus side (viral sequence of the junction) and the host side (host sequence of the junction) were globally aligned and scores calculated based on the Hamming distance. Since 50% of the junctions from the linear and Z-pattern integrations (MKL-1 L, BroLi L, PeTa L, UM-MCC-29 R+L, LoKe L, MKL-2 R, WoWe-2 R+L, UKE-MCC-1a L, UM-MCC-52 Chr5 R, MCC47 R) contained identical bases between virus and host at the virus-host junction and these bases cannot be definitely assigned to one or the other side, analysis was performed with both options (identical bases assigned to host or virus, respectively). No significant increase of scores from integration sites compared to scores obtained for 200 random viral and host sequences could be detected (p<0.05, dashed line). (B): Statistical analysis of homology stretches intercepted by non-matching sequences of variable length (gap homology, scores from all samples can be found in S1 Fig). Shown are P-values from comparison of scores from the virus and host side of samples showing Z-pattern or linear integration compared to scores obtained for 200 random viral and host sequences. Identical bases at the virus-host junctions were either assigned to the viral side (identical bases assigned to virus) or the host side (identical bases assigned to host). The virus side of Z-pattern integration shows significantly higher homology scores (p-value 0.0074; the dashed line marks the significance threshold of 0.05 at -log10 value 1.3). When identical bases were assigned to the host side no significant enrichment of homologies can be observed. (TIF) [file ppat.1008562.s006.tif]

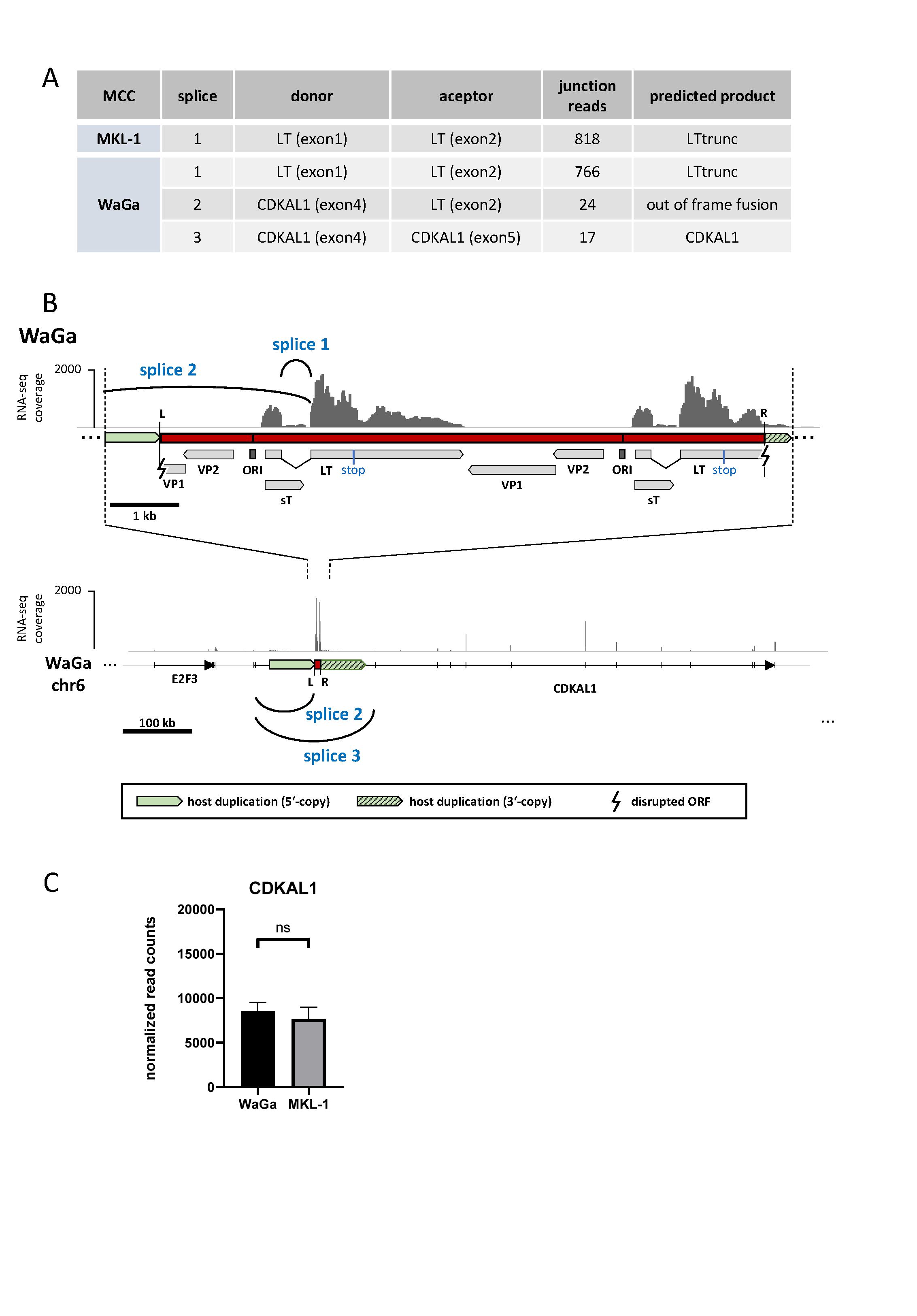

Supplement: S7 Fig — (A): Counts of host, virus and host-virus-fusion splice junction reads connecting to the splice acceptor of the second LT exon in MKL-1 and WaGa cells. In WaGa cells, we additionally counted splices between exons 4 and 5 of CDKAL1 (splice 3). It is likely that the transcripts harboring these splices originate from the copy of chr6 that does not contain the viral integrate. All detected splice events use annotated donor and acceptor sites as indicated. (B): RNA-Seq coverage at the integration locus. Detected splices are indicated by arcs. For further details, see legend to Fig 4. (C): Normalized RNA-Seq data of CDKAL1. Three RNA-Seq datasets of WaGa and MKL-1 (one dataset generated in this study and two datasets previously published [24]) were combined and subjected to standard DEseq2 analysis. Shown are Deseq2 normalized counts of CDKAL1 (n = 3, mean + SEM). The slight Log2 fold change of 0.15 between both cell lines was found to be not significant (ns) by DEseq2 analysis. (TIF) [file ppat.1008562.s007.tif]

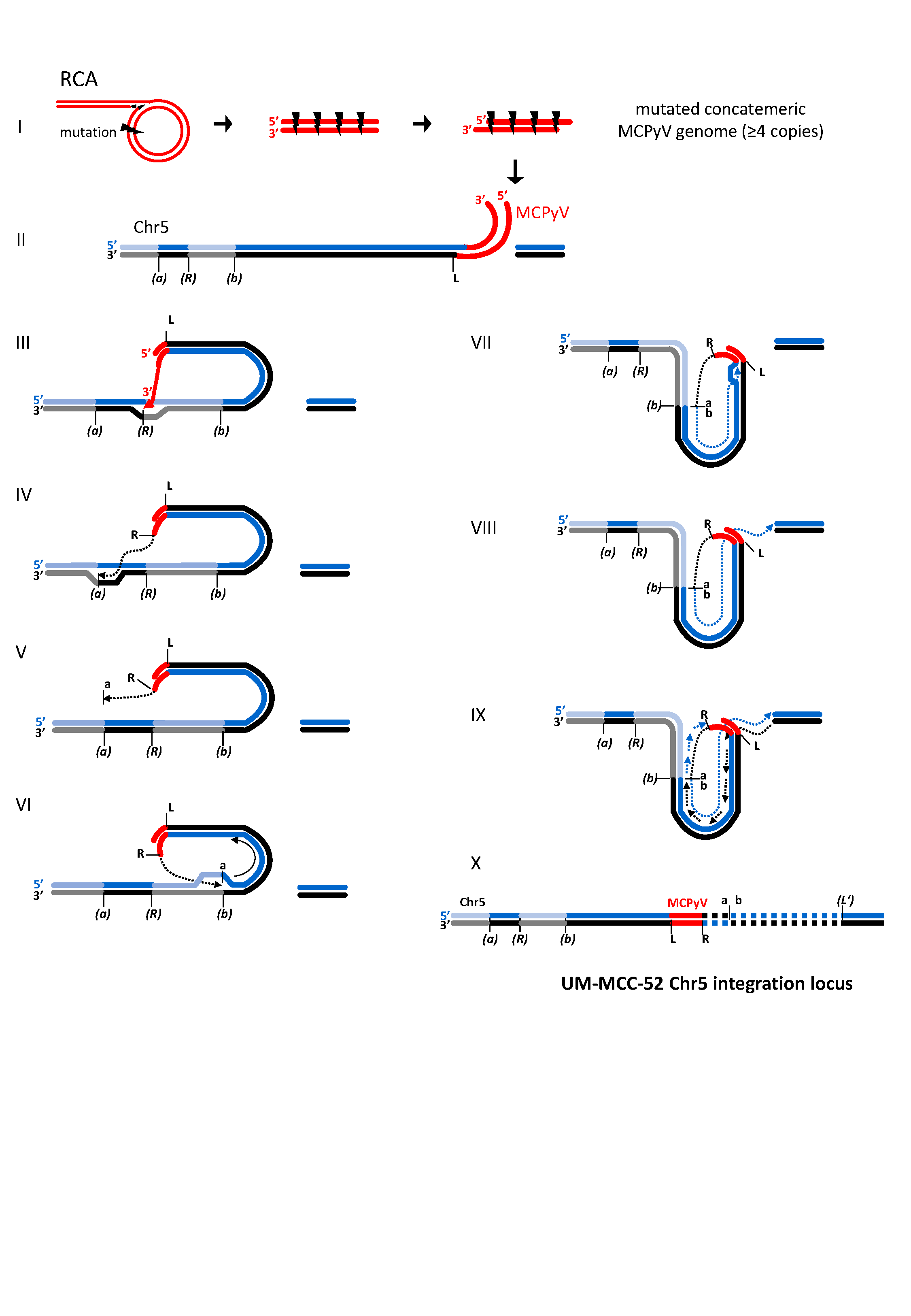

Supplement: S8 Fig — (I) Mutated concatemeric MCPyV genomes (at least 4 copies of MCPyV in this case) are produced by RCA and undergo 5‘resection by the host machinery. (II) Ligation to a ds break in the host DNA at the left side (L) is achieved by MMEJ. (III) The viral genome loops back and invades with its 3’ end a homologous host region and (IV) starts DNA synthesis in a D-loop structure (MMBIR). Different to the general model, the 3’ end of the viral genome aligns to the forward not the reverse strand. (V) DNA synthesis continues until it reaches site a and the D-loop disassembles. (VI) The newly synthesized strand invades again the host DNA (site b), this time aligning to the reverse strand. (VII) DNA replication can now proceed until it reaches L were it connects to the original ds break by an unknown mechanism (VIII). (IX) The complementary strand is synthesized in a conservative mode using the newly synthesized strand as a template. (X) For UM-MCC-52 the result is an amplification of several kbp of host sequence between L and b as well as an inverted sequence between site a and R. (TIF) [file ppat.1008562.s008.tif]
